# Large Language Models in Biochemistry Education: Comparative Evaluation of Performance

**DOI:** 10.2196/67244

**Published:** 2025-04-10

**Authors:** Olena Bolgova, Inna Shypilova, Volodymyr Mavrych

**Affiliations:** 1 College of Medicine, Alfaisal University, Al Takhassousi St, Riyadh, 11533, Saudi Arabia; 2 School of Medicine, St Mathews University, George Town, Cayman Islands

**Keywords:** ChatGPT, Claude, Gemini, Copilot, biochemistry, LLM, medical education, artificial intelligence, NLP, natural language processing, machine learning, large language model, AI, ML, comprehensive analysis, medical students, GPT-4, questionnaire, medical course, bioenergetics

## Abstract

**Background:**

Recent advancements in artificial intelligence (AI), particularly in large language models (LLMs), have started a new era of innovation across various fields, with medicine at the forefront of this technological revolution. Many studies indicated that at the current level of development, LLMs can pass different board exams. However, the ability to answer specific subject-related questions requires validation.

**Objective:**

The objective of this study was to conduct a comprehensive analysis comparing the performance of advanced LLM chatbots—Claude (Anthropic), GPT-4 (OpenAI), Gemini (Google), and Copilot (Microsoft)—against the academic results of medical students in the medical biochemistry course.

**Methods:**

We used 200 USMLE (United States Medical Licensing Examination)–style multiple-choice questions (MCQs) selected from the course exam database. They encompassed various complexity levels and were distributed across 23 distinctive topics. The questions with tables and images were not included in the study. The results of 5 successive attempts by Claude 3.5 Sonnet, GPT-4‐1106, Gemini 1.5 Flash, and Copilot to answer this questionnaire set were evaluated based on accuracy in August 2024. Statistica 13.5.0.17 (TIBCO Software Inc) was used to analyze the data’s basic statistics. Considering the binary nature of the data, the chi-square test was used to compare results among the different chatbots, with a statistical significance level of *P*<.05*.*

**Results:**

On average, the selected chatbots correctly answered 81.1% (SD 12.8%) of the questions, surpassing the students’ performance by 8.3% (*P=*.02). In this study, Claude showed the best performance in biochemistry MCQs, correctly answering 92.5% (185/200) of questions, followed by GPT-4 (170/200, 85%), Gemini (157/200, 78.5%), and Copilot (128/200, 64%). The chatbots demonstrated the best results in the following 4 topics: eicosanoids (mean 100%, SD 0%), bioenergetics and electron transport chain (mean 96.4%, SD 7.2%), hexose monophosphate pathway (mean 91.7%, SD 16.7%), and ketone bodies (mean 93.8%, SD 12.5%). The Pearson chi-square test indicated a statistically significant association between the answers of all 4 chatbots (*P*<.001 to *P*<.04).

**Conclusions:**

Our study suggests that different AI models may have unique strengths in specific medical fields, which could be leveraged for targeted support in biochemistry courses. This performance highlights the potential of AI in medical education and assessment.

## Introduction

Recent breakthroughs in artificial intelligence (AI), especially in large language models (LLMs), have started a new era of innovation across diverse fields, with medicine leading the charge in this technological revolution. The integration of AI into various medical disciplines such as oncology, radiology, and pathology has demonstrated its advancing clinical uses and its potential to revolutionize health care delivery [1-3]. As new LLMs continue to emerge and evolve, AI is poised to fundamentally reshape our understanding and approach to medicine, offering unprecedented opportunities for improved patient care, diagnostics, and medical education [4].

While academic interest in AI has surged in recent years, integrating AI technologies in educational settings, particularly medicine, has been uneven and fraught with challenges. Among many AI tools available, ChatGPT has emerged as a potential game-changer in medical education [5,6]. This sophisticated language model, powered by advanced neural networks, demonstrates a remarkable ability to interpret prompts and generate human-like responses, making it difficult to distinguish from human-produced language.

LLM’s underlying transformer architecture enables it to excel in natural language understanding, continuously processing and adapting to new information. This adaptability, combined with its vast knowledge base, presents promising opportunities for enhancing teaching and learning methodologies in medical education [7]. AI-powered tools such as ChatGPT may be particularly effective in addressing persistent challenges in student engagement, offering interactive and personalized learning experiences that traditional teaching methods often struggle to provide [8].

OpenAI’s GPT-4 and GPT-3.5, Google’s Gemini, and Anthropic’s Claude have emerged as frontrunners, offering unique capabilities and potential medical education and practice applications. As of 2024, the AI landscape in health care has become increasingly diverse, with over 20 LLMs available for public use. Among them, 4 are the most promising.

Anthropic developed Claude, an AI assistant known for its strong natural language understanding and generation capabilities. It has been trained on a wide range of data and is designed to be helpful, harmless, and honest. Claude has shown particular strength in tasks requiring nuanced understanding and ethical reasoning [9].

Created by OpenAI, GPT-4 is the latest GPT series iteration. It represents a significant advancement over its predecessor, GPT-3, with improved language understanding, generation, and reasoning capabilities. GPT-4 has demonstrated impressive performance across various domains, including coding, creative writing, and analytical tasks [10].

Developed by Google AI, Gemini is a multimodal AI model capable of understanding and generating text, images, and other forms of data. It comes in different sizes and is optimized for various tasks and computational requirements. Gemini has shown strong performance in complex reasoning tasks and can understand context across different modalities [11].

Created by GitHub in collaboration with OpenAI, Copilot is an AI pair programmer designed to assist developers by suggesting code completions and entire functions. It is now an integral part of Microsoft Windows. While primarily focused on coding tasks, Copilot’s underlying language model has shown capabilities in understanding and generating natural language [12].

One primary method for assessing the capabilities of LLMs in knowledge-based fields, including medicine, is their performance on multiple-choice tests [13-16]. The release of GPT-4 by OpenAI in 2023 marked a significant milestone, demonstrating impressive test-taking abilities across various domains [17]. Similarly, Claude 2 from Anthropic, released in June 2023, has gained attention for its ability to process larger input spaces (up to 100,000 tokens), potentially allowing for a more comprehensive analysis of medical texts and case studies [8].

The high accuracy demonstrated by ChatGPT-4 in answering multiple-choice questions (MCQs) compared to medical students’ performance is particularly noteworthy. It suggests that AI could be an effective study aid, helping students review and reinforce their knowledge across various medical subjects. However, it is essential to view AI as a complementary tool rather than a replacement for MCQs that have transformed from their conventional use as assessment tools to become a versatile educational approach in medical curricula. MCQs stimulate students’ cognitive abilities and promote active interaction with study materials. By using advanced generative AI-driven language models to address MCQs in medical physiology and other subjects, educators may provide students with an innovative and engaging learning experience, potentially enhancing their grasp of essential medical concepts, traditional teaching methods, or human expertise [18,19].

Recent studies have begun to compare the performance of different AI models in medical education contexts. For instance, Claude, an LLM developed by Anthropic, has shown promising results in solving medical MCQs. Some studies have indicated that Claude demonstrated a high frequency of right answers and explanations compared to ChatGPT-3.5 [8,20]. These comparative studies are crucial in understanding the strengths and limitations of different AI models in medical education. They help educators and researchers identify the most suitable tools for specific learning objectives and contexts within medical curricula.

Despite the promising results, it is important to note the variability in AI performance across different studies and question types. For example, while some studies reported high accuracy rates for ChatGPT in physiology tests [5,8], others found lower performance rates, particularly as the complexity and difficulty of questions increased [21,22]. This variability underscores the need for careful consideration when integrating AI tools into medical education. Educators must be aware of these tools’ strengths and limitations and ensure they are used appropriately to complement, rather than replace, traditional teaching methods.

It is important for educational strategies to prioritize the integration of LLMs into the curriculum as a vital aspect of the learning process. This integration should enable students to cultivate critical thinking and analytical skills, particularly in understanding the constraints of AI. LLMs have the potential to offer students in-depth knowledge and diverse viewpoints, facilitating a more thorough comprehension of intricate medical concepts [23]. By using the output of LLMs and working alongside educators to draw upon their existing knowledge, students can actively participate in the learning process. This collaborative approach allows for the refinement of their understanding and insights. The future of medical education depends on the seamless integration of human expertise with AI-powered tools [3,19,23].

The aim of this study was to conduct a comprehensive analysis comparing the performance of advanced LLM chatbots—GPT-4, Claude, Copilot, and Gemini—against the academic results of medical students in biochemistry. The research objectives were to evaluate the following hypotheses:

- The AI chatbots will perform similarly to medical students on factual recall and basic concept application questions in biochemistry but may show differences in performance on complex problem-solving scenarios.
- There will be significant variation in performance among the different AI models, with newer models (GPT-4 and Claude) potentially showing higher accuracy compared to earlier versions.
- The AI-driven LLMs’ performance will vary across different biochemistry topics, with potentially stronger performance in areas requiring systematic pathway analysis and weaker performance in topics requiring integration of clinical context.

## Methods

### Study Design

This study focused on a comparative analysis of the capabilities of different AI-driven LLMs in the medical biochemistry course. The research included an examination of 4 chatbots currently available to the public: Claude (Anthropic), GPT-4 (OpenAI), Gemini (Google), and Copilot (Microsoft).

A total of 200 scenario-based MCQs with 4 options and a single correct answer were randomly chosen from the medical biochemistry course’s examination database for medical students and validated by 2 independent experts. The study did not include questions with images and tables. The selected questions encompassed various levels of complexity. They were distributed across 23 distinctive categories: structural proteins and associated diseases, globular proteins and hemoglobin, red blood cells (RBCs) and anemia, structure and function of amino acids, structure and function of proteins, bioenergetics and electron transport chain, enzymes, glycolysis and gluconeogenesis, glycogen, signaling mechanisms, pyruvate dehydrogenase and Krebs cycle, cholesterol metabolism, eicosanoids, fatty acid metabolism, fructose and galactose metabolism, hexose monophosphate pathway, ketone bodies, lipoproteins, lysosomal storage diseases, amino acid metabolism, fast and fed state, heme metabolism, and nitrogen metabolism.

### Data Collection

For the testing phase, each selected chatbot was required to answer a set of 200 questions, and their performance was evaluated against the responses provided by medical students for the same set of questions. Claude 3.5 Sonnet, GPT-4‐1106, Gemini 1.5 Flash, and Copilot proficiency in responding to MCQs was assessed in the last 2 weeks of August 2024. An OpenAI paid subscription was obtained to get GPT-4 access.

Each chatbot was given the prompt “generate the list of correct answers for the following MCQs” and provided with a first set of 50 questions; following with the same prompt and 3 more sets of 50 MCQs each, totally there were 200 MCQs in the questionnaire. After that, this procedure was repeated 5 times (no time period between the attempts was assigned). The results of 5 successive attempts by each chatbot to answer this questionnaire set were meticulously recorded in a Microsoft Excel spreadsheet and evaluated based on accuracy. A total of 4000 answers from LLMs were analyzed.

Five random answers were generated and analyzed for the same MCQ set using the RAND() function in Excel (Microsoft 365) to compare chatbot results with random guessing.

### Data Analysis

The answers provided by each LLM were recorded and input into the Excel spreadsheet (Microsoft 365). The data from each (1-5) attempt was matched with the answer key and compared with all previous attempts, finding the percentage of repeated and correct answers among them. After that, a detailed item analysis was performed for each chatbot concerning different question categories.

Statistica 13.5.0.17 (TIBCO Software) was used to analyze the data’s basic statistics. Considering the data’s binary nature, the chi-square test was used to compare results among the different chatbots.

## Results

### Overview

According to our data, on average, 4 selected chatbots accurately answered 81.1% (SD 12%) of 200 MCQs from the medical biochemistry course. This result was 8.3% (*P=*.02) above the students’ average (mean 72.8%, SD 12.7%) and almost 4 times better than randomly generated responses (mean 22%, SD 2.9%) for the same questions.

**Figure 1.**
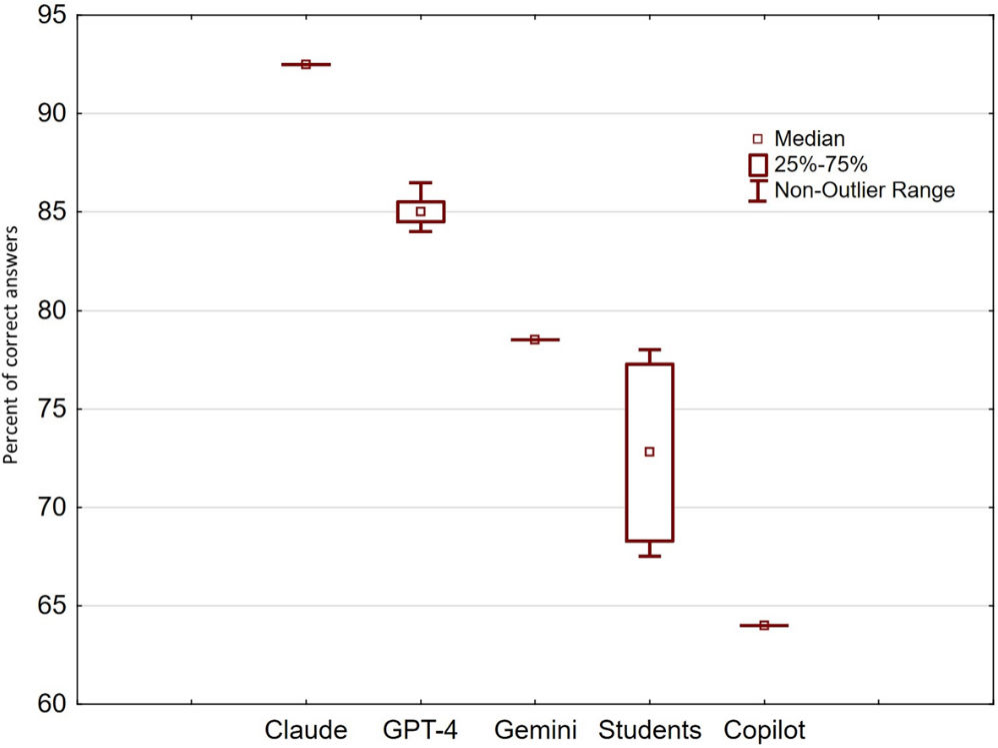
Percentile of correct answers from different chatbots and students on 200 multiple-choice questions from the medical biochemistry course.

There was a significant variation in correct responses among the chatbots. The best result was recorded for Claude (92.5%, SD 0%), followed by GPT-4 (mean 85.1%, SD 1%) and Gemini (mean 78.5%, SD 0%), which were better than the students’ average. Copilot showed the lowest result (mean 64%, SD 0%; Figure 1).

Interestingly, all chatbots answered 104 (52%) of the 200 questions correctly in all attempts. General item analysis revealed that eicosanoids, bioenergetics and electron transport chain, hexose monophosphate pathway, and ketone bodies were the 4 best topics, with the mean (SD) results for all chatbots being 100% (0%), 96.4% (7.2%), 91.7% (16.7%), and 93.8% (12.5%), respectively.

In contrast, the lowest results were recorded for globular proteins and hemoglobin (mean 58.4%, SD 26.4%), lipoproteins (mean 64.6%, SD 20.3%), and fructose and galactose metabolism questions (mean 65.8%, SD 29.9%).

**Figure 2.**
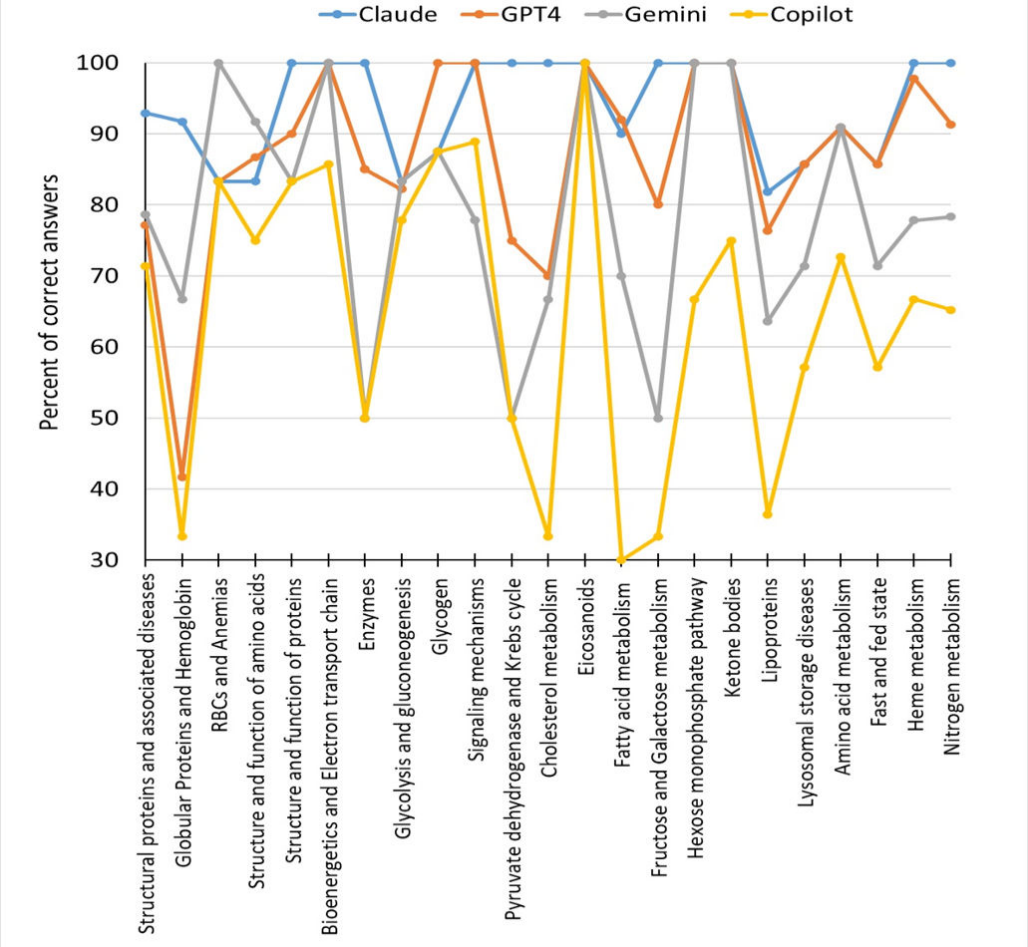
Evaluation of chatbot performance in different topics of the medical biochemistry course. RBC: red blood cell.

After that, each chatbot’s results for all 23 topics were evaluated (Figure 2).

### Claude

Claude, offered by Anthropic, provided 92.5% (185/200) correct answers to the set of biochemistry MCQs. The answers in this second and all subsequent attempts were identical to the first. As the chatbot claims, its knowledge base has not changed between attempts, and it applies the same reasoning to answer each question. It was the best result among the 5 chatbots, 19.7% better than the student average and 70.5% superior to random guessing. The item analysis suggested that Claude correctly answered all questions (100%) from the following 12 categories: structure and function of proteins, bioenergetics and electron transport chain, enzymes, signaling mechanisms, pyruvate dehydrogenase and Krebs cycle, cholesterol metabolism, eicosanoids, fructose and galactose metabolism, hexose monophosphate pathway, ketone bodies, heme metabolism, and nitrogen metabolism. The lowest result (81.8%) was recorded for the lipoproteins. For the rest of the topics, the percentile of correct answers was 83.3%‐91.7%.

Claude did not solve 15 (7.5%) out of 200 MCQs from the entire questionnaire set. These were comprehensive questions about RBCs, hemoglobin, enzymes, biotin deficiency, and lipoproteins.

### GPT-4

The results of 5 successive ChatGPT-4 (OpenAI) attempts to answer the set of 200 biochemistry MCQs showed 85.1% (SD 1%) correct answers on average. The best result of its 5 attempts was 86.5%, 13.7% better than the average for medical students and 64.5% above random guessing. The fourth attempt was the most successful; the mean results of the other 4 attempts were close to 85% (range 84%‐85.5%). The coincidence generated by GPT-4 answers with the previous attempts was 91.5%‐94.5%, and the coincidence of correct answers among them was in the range 81%‐83.6%.

Of the 200 questions, 158 (79%) were answered correctly across all 5 attempts and considered a solid knowledge area for GPT-4. Most of these MCQs were recall questions, but some were complex and required critical thinking. The item analysis indicated that the best 6 categories with 100% correct answers were bioenergetics and electron transport chain, glycogen, signaling mechanisms, eicosanoids, hexose monophosphate pathway, and ketone bodies. The lowest result was recorded for globular proteins and hemoglobin questions—only 41.7% of the correct answers. For the rest of the topics, the percentile of correct answers was 77.1%‐97.8%.

GPT-4 did not answer 17 (8.5%) of the 200 MCQs from the entire questionnaire set in any 1 out of all 5 attempts. These were more comprehensive questions about defective proteins, oxygen saturation, anemia, amino acids, glycogen, glycolysis and gluconeogenesis, and lipoproteins.

### Gemini

Google recently introduced Gemini as a successor to Bard. The results of 5 attempts by Gemini to answer the set of 200 biochemistry MCQs showed 157 (78.5%) correct answers, 5.7% above the average for medical students and 56.5% above the random answers. Unlike Bard, 5 successive attempts from Gemini were similar; the same answers were received.

The item analysis of these 157 correct answers shows that Gemini did the best (100% accurate) for questions in the following 5 categories: RBCs and anemia, bioenergetics and electron transport chain, eicosanoids, hexose monophosphate pathway, and ketone bodies. Most of these MCQs were recall questions. The lowest 50% results were recorded for the following 3 categories: enzymes, pyruvate dehydrogenase and Krebs cycle, and fructose and galactose metabolism. Gemini’s responses in other topics were in the 63.6%‐91.7% interval. Gemini did not answer 43 (21.5%) of the 200 MCQs from the entire questionnaire set, which were comprehensive questions mostly about proteins, enzymes, the Krebs cycle, fatty acids, fructose, and galactose metabolism.

### Copilot

Microsoft’s Copilot can accept only up to 2000 characters in the prompt, so only 2 to 7 MCQs can be answered at a time, which is inconvenient to work with. The results received on the first try were not different from 4 successive attempts, so there was zero variation among all 5 attempts. Copilot generated 128 (64%) accurate answers for the same set of 200 MCQs from the biochemistry course, 8.8% lower than the average medical student but 42% better than random guessing.

The item analysis of these 126 correct answers indicated that these MCQs were mostly recall questions. The best result was shown for the eicosanoids category (100%), and the lowest was for fatty acid metabolism (only 30% of correct answers). Copilot’s responses in other topics vary from 33.3% to 88.9%. Copilot did not answer 72 (36%) of the 200 MCQs from the questionnaire set. These questions concerned proteins, hemoglobin, amino acids, enzymes, fatty acids, pyruvate dehydrogenase, Krebs cycle, and fast and fed state.

### Pearson Chi-Square Test Results

Table 1 shows the results of the Pearson chi-square test, which we used due to the binary nature of the data to compare the performance of the different AI-driven chatbots against each other.

The null hypothesis was rejected because the *P* value for all chatbots was less than α (*P=*.05), so there is a statistically significant association between the answers of all 4 chatbots.

**Table 1.** Pearson chi-square test results to compare the performance of Claude, GPT-4, Gemini, and Copilot against each other.

Large language models	Chi-square (*df*)	*P* value
Claude × GPT-4	19.7 (1)	<.001
Claude × Gemini	6.1 (1)	.01
Claude × Copilot	4.1 (1)	.04
GPT-4 × Gemini	33.1 (1)	<.001
GPT-4 × Copilot	15.9 (1)	<.001
Gemini × Copilot	23.5 (1)	<.001

## Discussion

### Principal Findings

Medical education is rapidly evolving, with AI playing an increasingly significant role. In this context, evaluating AI efficacy and relevancy to results is crucial, particularly given the precision and depth of understanding required in medical practice. AI-driven LLMs such as ChatGPT, Claude, Copilot, and Gemini have been compared against medical students in various studies, revealing both the strengths and limitations of AI in medical education. These comparisons show how AI can enhance human learning while also highlighting areas where it may not measure up. Research into AI’s role in medical training has uncovered intriguing possibilities and important constraints [1,5,7].

MCQs form a cornerstone of assessment in medical education. Analyzing these questions is vital as it allows educators to assess their effectiveness in testing higher-order thinking and clinical reasoning skills, ensuring that assessments accurately reflect the competencies required for medical practice [18]. While LLMs have demonstrated impressive capabilities in answering queries and simulating scenarios, the depth and breadth of their understanding, particularly concerning MCQs in medical exams, still requires thorough evaluation [19].

The comparative analysis of LLMs and medical students in biochemistry assessment reveals several intriguing patterns that both confirm and challenge our initial hypotheses. While we anticipated comparable performance between AI models and medical students, the results demonstrated that LLMs not only matched but significantly exceeded student performance, with an 8.3% higher average score (*P=*.02) across 200 medical biochemistry questions. This finding particularly supports our hypothesis regarding factual recall and concept application, though with a more pronounced advantage for AI systems than initially predicted. The observed variation in performance among different LLM platforms—ranging from Claude’s exceptional 92.5% (185/200) accuracy to Copilot’s more modest 64% (128/200)—aligns with our hypothesis about performance differences between AI models, suggesting that architectural and training differences significantly impact their capabilities in specialized medical knowledge domains.

### Comparison to Literature

Recent studies have shown that LLMs, specifically GPT-4, often outperform medical students on MCQ items in board and licensing exams. This finding underscores the significance of MCQs in medical licensing exams, extensively used in crucial assessments worldwide. Examples include the Peruvian National Licensing Medical Examination, the United States Medical Licensing Examination (USMLE), the United Kingdom Medical Licensing Assessment (UKMLA), and the Australian Medical Council (AMC) Exam [20,24-26]. The widespread use of MCQs is attributed to their effectiveness in evaluating higher-order skills through complex clinical scenarios, analysis, and problem-solving. These questions assess students’ ability to integrate information, reflecting real-world challenges and shaping competent professionals. It is well correlated with the results of our study, which have shown that the selected 4 chatbots answered correctly to 81.1% (SD 12%) of the 200 questions from the medical biochemistry course, which is 8.3% above the students’ average.

Another comprehensive study compared the results of 4 LLMs across 163 questions from sample NBME (National Board of Medical Examiners) clinical subject exams. The results were striking: GPT-4 achieved a perfect score of 100% (163/163), significantly outperforming GPT-3.5, Claude, and Bard. GPT-3.5 scored 82.2% (134/163), Claude 84.7% (138/163), and Bard 75.5% (123/163). The statistical superiority of GPT-4 was evident, with no significant differences observed among the other 3 models [27]. Interestingly, while GPT-4 excelled across all subject exams, the different models demonstrated variable strengths. GPT-3.5 performed best in family medicine and obstetrics and gynecology, Claude in surgery, and Bard in surgery and neurology. The surgery exam yielded the highest average score across all models, while family medicine had the lowest. GPT-4’s exceptional performance may be attributed to its extensive training data, which exceeded 45 terabytes by September 2021, despite not being specifically fine-tuned for medical data [10].

Our data contradict this clinical study and suggest that GPT-4 did well with 85% (170/200) of correct answers but is not currently the most proficient chatbot for biochemistry questions. The best result was recorded for Claude, with an impressive 92.5% (185/200) of the correct answers. Gemini took third place with 78.5% (157/200) of correct answers, which is still above the student’s average of 72.8% (SD 5.2%) for the same questions. The lowest result was recorded for Copilot (128/200, 64%).

These findings highlight the potential of LLMs in medical education and practice. Their ability to tackle complex medical questions opens doors to innovative clinical decision support, research, and education applications. However, it is worth noting that GPT-4, the only LLM in this study not available for free, could be less accessible to a broad range of students, potentially limiting its widespread use in educational settings.

Several studies have evaluated ChatGPT’s performance in biochemistry. One study examined GPT-3.5’s potential as a self-study adjunct for medical students in biochemistry, using 200 questions. ChatGPT provided correct answers to 58% (116/200) of the biochemistry questions. While this performance allowed it to pass the university’s medical biochemistry exam, the study suggests there is room for improvement in GPT-3.5 as a comprehensive and reliable self-learning tool [28].

Another study focused on ChatGPT’s ability to address higher-order questions in medical biochemistry. Using GPT-3.5, researchers conducted a web-based cross-sectional study presenting 200 randomly selected, complex reasoning questions from an institutional question bank, classified according to CBME (Competency-Based Medical Education) curriculum modules. Two expert biochemistry academicians evaluated responses on a 0‐5 scale. The AI achieved a median score of 4 (IQR 3.5-4.5), which was comparable to a hypothetical value of 4 (*P*=.16) but significantly lower than the maximum of 5 (*P*=.001). These results suggest that GPT-3.5 shows promise as an effective tool for addressing complex questions in medical biochemistry, demonstrating its potential in handling higher-order thinking tasks in this field [29].

Our research confirms that GPT-4 has significant improvements and is superior to GPT-3.5. Our data suggest that GPT-4 responded correctly to 84%‐86.5% of MCQs, and 79% answered correctly across all 5 attempts.

### Implications of Findings

The implications of AI’s performance in medical education extend beyond mere test-taking abilities. LLMs can answer complex medical questions that raise important questions about the future of medical education and topics in which LLMs demonstrate proficiency, so they may be used to assist students. The detailed analysis of MCQs in our study revealed that questions from 4 topics are well answered by all chatbots: eicosanoids, bioenergetics, electron transport chain, and ketone bodies. In contrast, the lowest results were recorded for globular proteins and hemoglobin, lipoproteins, and fructose and galactose metabolism questions. However, there was a significant difference in the 4 LLMs performances. Claude showed the most impressive results and answered all questions (100%) from 12 categories: structure and function of proteins, bioenergetics and electron transport chain, enzymes, signaling mechanisms, pyruvate dehydrogenase and Krebs cycle, cholesterol metabolism, eicosanoids, fructose and galactose metabolism, hexose monophosphate pathway, ketone bodies, heme, and nitrogen metabolism.

In conclusion, the rapid advancements in AI technology, particularly in medical education, present opportunities and challenges. While LLMs have shown impressive capabilities in answering medical exam questions, it is crucial to remember that medical education encompasses more than just knowledge acquisition. Clinical skills, empathy, ethical decision-making, and the ability to navigate complex health care systems are all integral parts of medical training that current AI models may not fully capture.

As we progress, we must continuously evaluate AI’s role in medical education, ensuring that it complements rather than replaces human expertise. Our findings also have important implications for assessment strategies in medical education. The ability of LLMs to surf the net and do better than medical students on MCQ-based evaluation is an assault on the traditional ways of measuring medical performance and calls for a better understanding of how medical knowledge and skills should be assessed. While such results provide ideas on how to develop a curriculum and manage educational resources, they also highlight the need to ensure that the value of AI in measuring certain aspects of medical training, such as clinical reasoning, interaction with patients, and even decision-making ethics, is always respected. This underscores the need for medical education to continue emphasizing the development of comprehensive clinical skills beyond what can be measured through standardized testing.

### Future Directions

Future research in this field should pursue several key routes to better understand and implement AI technologies in medical education. Long-term studies are needed to evaluate the impact of LLM integration on student learning outcomes, particularly focusing on how AI-assisted learning affects knowledge retention, clinical reasoning development, and overall academic performance. These studies should incorporate diverse assessment methods beyond MCQs, including case-based scenarios, open-ended questions, and practical clinical applications of biochemistry knowledge across different medical disciplines to understand whether the observed performance patterns are consistent.

### Strengths and Limitations

This study represents one of the first comprehensive comparisons between multiple leading LLMs and medical students in the specific context of medical biochemistry education. The large sample size of 200 questions provided a robust dataset for analysis, covering a broad spectrum of biochemistry topics typically encountered in medical education. The inclusion of multiple LLM platforms (GPT-4, Claude, Copilot, and Gemini) allowed for a nuanced comparison of AI capabilities across different models, providing valuable insights into their relative strengths and potential applications in medical education.

Several limitations should be considered when interpreting these results. This study’s findings on different chatbot proficiencies are limited to MCQs from the biochemistry course, which may not represent other medical questions or contexts. In addition, the sample size of 200 questions, excluding questions with images or tables, may not capture the full range of difficulty levels or content areas.

LLMs receive regular updates, which result from training on inputs and tuning so that they may provide different answers depending on the testing date. Another limitation is that GPT-4, which performed well, is not freely available, potentially limiting its applicability in widespread educational settings.

### Conclusions

LLMs such as ChatGPT, Claude, Copilot, and Gemini have impressive capabilities in answering MCQs, often outperforming medical students. In this study, the selected chatbots outperformed students’ results. These findings highlight the potential of AI in medical education and assessment. Different LLMs exhibit varying strengths in different topics of medical biochemistry courses. In this study, Claude showed the best performance, followed by GPT-4, Gemini, and Copilot. This variability suggests that different AI models may have unique strengths in specific medical fields, which could be leveraged for targeted educational support. The strong performance of LLMs in answering complex medical questions raises important considerations for the future of medical education. While AI demonstrates proficiency in knowledge-based assessments, it is crucial to remember that medical training encompasses more than just information recall. Clinical reasoning, empathy, ethical decision-making, and navigating health care systems remain essential components that current AI models may need to capture fully.

## Abbreviations

AI: artificial intelligence
AMC: Australian Medical Council
CBME: Competency-Based Medical Education
LLMs: large language models
MCQ: multiple-choice question
NBME: National Board of Medical Examiners
RBC: red blood cell
UKMLA: United Kingdom Medical Licensing Assessment
USMLE: United States Medical Licensing Examination
